# Target Region Selection Is a Critical Determinant of Community Fingerprints Generated by 16S Pyrosequencing

**DOI:** 10.1371/journal.pone.0020956

**Published:** 2011-06-29

**Authors:** Purnima S. Kumar, Michael R. Brooker, Scot E. Dowd, Terry Camerlengo

**Affiliations:** 1 Division of Periodontology, College of Dentistry, The Ohio State University, Columbus, Ohio, United States of America; 2 Research Testing Laboratory, Lubbock, Texas, United States of America; 3 Comprehensive Cancer Center, The Ohio State University, Columbus, Ohio, United States of America; J. Craig Venter Institute, United States of America

## Abstract

Pyrosequencing of 16S rRNA genes allows for in-depth characterization of complex microbial communities. Although it is known that primer selection can influence the profile of a community generated by sequencing, the extent and severity of this bias on deep-sequencing methodologies is not well elucidated. We tested the hypothesis that the hypervariable region targeted for sequencing and primer degeneracy play important roles in influencing the composition of 16S pyrotag communities. Subgingival plaque from deep sites of current smokers with chronic periodontitis was analyzed using Sanger sequencing and pyrosequencing using 4 primer pairs. Greater numbers of species were detected by pyrosequencing than by Sanger sequencing. Rare taxa constituted nearly 6% of each pyrotag community and less than 1% of the Sanger sequencing community. However, the different target regions selected for pyrosequencing did not demonstrate a significant difference in the number of rare and abundant taxa detected. The genera *Prevotella*, *Fusobacterium*, *Streptococcus*, *Granulicatella*, *Bacteroides*, *Porphyromonas* and *Treponema* were abundant when the V1–V3 region was targeted, while *Streptococcus*, *Treponema*, *Prevotella*, *Eubacterium*, *Porphyromonas*, *Campylobacer* and *Enterococcus* predominated in the community generated by V4–V6 primers, and the most numerous genera in the V7–V9 community were *Veillonella*, *Streptococcus*, *Eubacterium*, *Enterococcus*, *Treponema*, *Catonella* and *Selenomonas*. Targeting the V4–V6 region failed to detect the genus *Fusobacterium*, while the taxa *Selenomonas*, *TM7* and *Mycoplasma* were not detected by the V7–V9 primer pairs. The communities generated by degenerate and non-degenerate primers did not demonstrate significant differences. Averaging the community fingerprints generated by V1–V3 and V7–V9 primers providesd results similar to Sanger sequencing, while allowing a significantly greater depth of coverage than is possible with Sanger sequencing. It is therefore important to use primers targeted to these two regions of the 16S rRNA gene in all deep-sequencing efforts to obtain representational characterization of complex microbial communities.

## Introduction

Molecular approaches have revealed the presence of large numbers of as-yet-uncultivated organisms in the subgingival microbiome; creating a paradigm shift in our understanding of periodontal health and disease [1], [2], [3], [4]. In recent years, sequencing of 16S rRNA genes by the Sanger method (16S cloning and sequencing) has been widely used to examine subgingival microbial profiles in periodontal health and disease, as well as to characterize compositional shifts in these communities [5], [6], [7], [8], [9]. However, recent studies suggest that next-generation sequencing methodologies provide an economical and significantly higher-throughput alternative to Sanger sequencing for comparative genomics [10], [11].

Pyrosequencing of PCR-amplified 16S rDNA (‘16S pyrotags’) is a next-generation sequencing methodology that is capable of generating thousands of sequences from several samples simultaneously. The unprecedented sampling depth provided by this deep-sequencing approach allows the identification of several numerically minor or rare species within a community and has revealed a significantly greater level of microbial diversity than was previously apparent with Sanger sequencing [12], [13].

Unlike Sanger sequencing, which is capable of sequencing the entire gene, pyrosequencing is currently limited to generating sequences that are usually 350–500 bp in length. In order to improve community coverage, various investigations have employed primers that target different regions of the gene [12], [13], [14]. It has previously been shown, using Sanger sequencing, that the region of the 16S gene that is targeted for sequencing as well as the degeneracy of the sequencing primers introduce a level of bias into the community profile [2], [15]. Since pyrosequencing provides an enormously increased depth-of-coverage, it is important to understand the extent and severity of bias introduced by primer selection on the profile of any given community.

Previous studies have examined this bias using simulated datasets obtained by truncating full-length sequences, *in silico* testing of primer sequences for community coverage rates or by analyzing artificial bacterial communities created by mixing bacterial isolates [15], [16], [17], [18], [19]. However, it is logical to expect that fragment length (∼1.5 kb with Sanger sequencing and 150–500 bp with pyrosequencing) and as well as sequencing chemistry will affect amplification efficiency; therefore, profiles derived from artificially generated sequences may not accurately represent the coverage obtained from naturally occurring microbial communities. In fact, a recent investigation comparing 454 and Illumina sequencing has found significant divergence between *in silico* predictions and experimental results, emphasizing the need for experimental validation of primer pairs [20]. Hence, it is important to investigate the extent of this bias using sequences derived from clinical samples.

The purpose of this investigation, therefore, was to examine the bias introduced by target region selection and as well as by primer degeneracy on coverage of subgingival microbial communities using pyrosequencing.

## Methods

### Subject selection

Approval for this study was obtained from the Office of Responsible Research Practices at The Ohio State University. 10 current smokers with generalized moderate to severe chronic periodontitis were identified following clinical and radiographic examination and written informed consent was obtained. Exclusion criteria included diabetes, HIV infection, use of immunosuppressant medications, bisphosphonates or steroids, antibiotic therapy or oral prophylactic procedures within the last three months and less than 20 teeth in the dentition.

### Sample collection and DNA isolation

Subgingival plaque samples were collected and pooled from four non-adjacent proximal sites demonstrating at least 6 mm of attachment loss and 5 mm of probe depths. Samples were collected by inserting 4 sterile endodontic paper points (Caulk-Dentsply) into each of the 4 sites for 10 seconds, following isolation and supragingival plaque removal. Samples were placed in 1.5 ml microcentrifuge tubes and frozen until further analysis. Bacteria were separated from the paper points by adding 200 µl of phosphate buffered saline (PBS) to the tubes and vortexing. The points were then removed, and DNA was isolated with a Qiagen DNA MiniAmp kit (Qiagen, Valencia, CA) using the tissue protocol according to the manufacturer's instructions.

### Selection and optimization of primers

Four sets of primers were used to amplify each sample (A17 and 519R, 27F and 515R, 519F and 1114R, 1114F and 317). The primer sequences are listed in Table 1. Primer pairs were selected to generate 400–500 bp products from contiguous regions of the 16S rRNA gene. Previous sequencing-based investigations were examined and the primers most commonly used in these studies were selected [2], [6], [7], [8], [9], [13], [21], [22]. The universality of the primer pairs was assessed by comparing them to our locally hosted, curated database of 1800 nearly full-length 16S sequences derived from GenBank. MacVector was used for alignment and determining melting temperatures and GC ratios of the resulting amplicons. Complementary sequences were generated from the published sequences of primers 519 and 1114. Degeneracies were added to primer 515R following comparison to the oral bacterial database to maximize matches of primer against bacterial sequences.

**Table 1:** Sequences of primers used in study.

Target region	Primer name (reference)	Primer sequence	% GC ratio	
			Primer	Product
V1–V3	A17 (Kumar et al 2005)	5′- GTT TGA TCC TGG CTC AG- 3′	52.9	53.4
	519R (Lane et al 1991)	5′- GTA TTA CCG CGG CAG CTG GCA C-3′	63.6	
V1–V3	27F (Lane et al 1991)	5′- AGA GTT TGA TGM TGG CTC AG-3′	50	53.4
	515R (modified from Kroes et al 1999)	5′- TTA CCG CGG CMG CSG GCA C-3′	78.9	
V4–V6	519F(modified from Lane et al 1991)	5′- GTG CCA GCT GCC GCG GTA ATA C-3′	63.6	54.6
	1114R( modified from Stackebrandt and Goodfellow 1991)	5′- GGG TTG CGC TCG TTG C-3′	68.8	
V7–V9	1114F(Stackebrandt and Goodfellow 1991)	5′- GCA ACG AGC GCA ACC C-3′	68.8	54.2
	317 (Kumar et al 2005)	5′- AAG GAG GTG ATC CAG GC-3′	58.8	
Sanger	A17 (Kumar et al 2005)	5′- GTT TGA TCC TGG CTC AG- 3′	52.9	53.8
	317 (Kumar et al 2005)	5′- AAG GAG GTG ATC CAG GC-3′	58.8	

### Pyrosequencing

Multiplexed bacterial tag-encoded FLX amplicon pyrosequencing (bTEFAP) was performed using the Titanium platform (Roche Applied Science, Indianapolis, IN) as previously described [22] in a commercial facility (Research and Testing Laboratories, Lubbock, TX). Briefly, a single step PCR with broad-range universal primers and 22 cycles of amplification was used to amplify the 16S rRNA genes as well as to introduce adaptor sequences and sample-specific 10-mer oligonucleotide tags into the DNA. The same bar codes were utilized for each primer set. Three regions of the 16S gene were sequenced from each sample (V1–V3, V4–V6, V7–V9). Adaptor sequences were trimmed from raw data with 98% or more of bases demonstrating a quality control of 30 and sequences binned into individual sample collections based on bar-code sequence tags, which were then trimmed. The resulting files were denoised with Pyronoise [23] and depleted of chimeras using B2C2 (http://www.researchandtesting.com/B2C2.html). Sequences less than <300 bp in length were deleted and the rest were clustered into species-level operational taxonomic units (s-OTUs) at 97% sequence similarity and assigned a taxonomic identity by alignment to locally hosted version of the Greengenes database [24] using the Blastn algorithm. Phylogenetic trees were generated and visualized using FastTree [25]. All analyses were conducted within the virtual environment provided by the QIIME pipeline [26].

### Statistical analysis

Species-level OTUs (s-OTUs) were used to compute the Shannon Diversity and Equitability indices for each sample. EstimateS ((Version 7.5, R. K. Colwell, http://purl.oclc.org/estimates) was used to compute the indices and statistical analyses were carried out with JMP (SAS Institute Inc., Cary, NC). The indices were compared between groups using ANOVA. A variance stabilizing transformation was used to create normal distribution of the data as previously described [27], [28]. Two sample t-tests were used to compare the transformed values of species and genus-level OTUs between groups. Fisher's exact test was used to test for presence or absence of genera.

## Results

The pyrotag sequences were compared to previously published data obtained by Sanger sequencing using the primer pairs A17 and 317 on the same samples [27]. A subset of the pyrosequencing data was created using a random number generator to select 100 pyrotag sequences from each primer set. This subset was compared to an equivalent number of Sanger sequences. A total of 1054 nearly full-length sequences (1300–1460 bp) were identified by Sanger sequencing, and 167,210 sequences by pyrosequencing, representing a 167-fold increase in depth-of-coverage with pyrosequencing.

**Figure 1:**
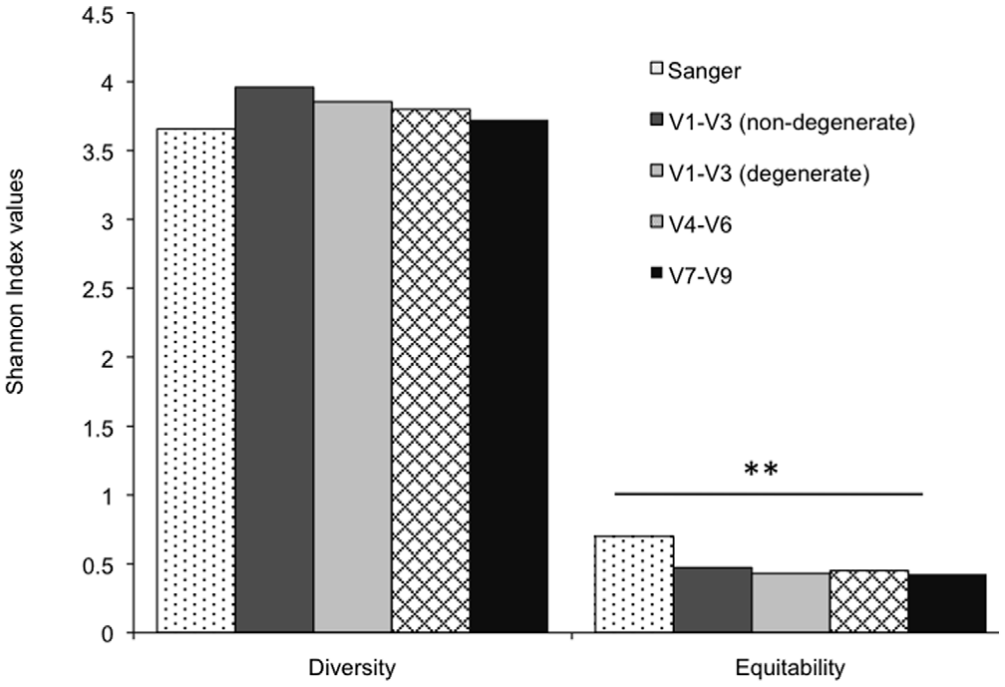
Shannon diversity and equitability indices of pyrotag and Sanger communities. No differences were detected between any of the pyrotag communities; however, the Sanger community demonstrated significantly greater equitable than all the pyrotag communities (** p<0.01, ANOVA).

Figure 1 shows the Shannon Diversity and Equitability indices for all primer sets. The Diversity Index was not different between groups; however, the community generated by Sanger sequencing demonstrated significantly greater equitability than all the pyrotag communities (p<0.01, ANOVA). Pyrotag communities generated by the 4 primer pairs demonstrated similar diversity.

**Figure 2:**
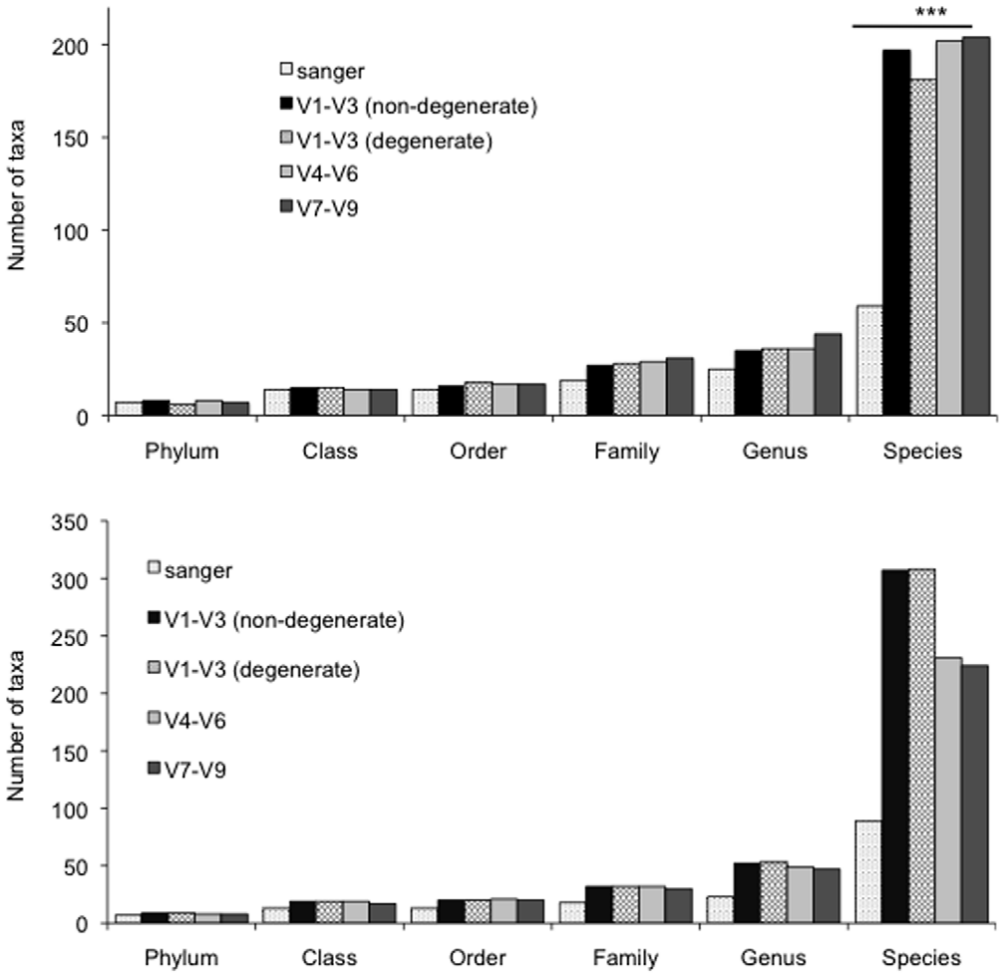
Distribution of sequences by taxa. Rare taxa are shown in Figure 2A and abundant taxa in Figure 2B. The Sanger community demonstrated significantly fewer species-level taxa than pyrosequencing (*** p<0.001, ANOVA). There were no differences between the pyrotag sequences.

Figures 2A and 2B show the distribution of rare and abundant taxa by primer pair and sequencing methodology. 1.9% of sequences could not be classified into any taxon below the level of domain. Taxa with less than 20 overall sequences were designated as rare. Sanger sequences demonstrated significantly lower coverage of rare as well as abundant species than pyrosequencing (p<0.001, ANOVA). However, there were no differences in the number of rare and abundant taxa in any of the pyrotag communities.

**Figure 3:**
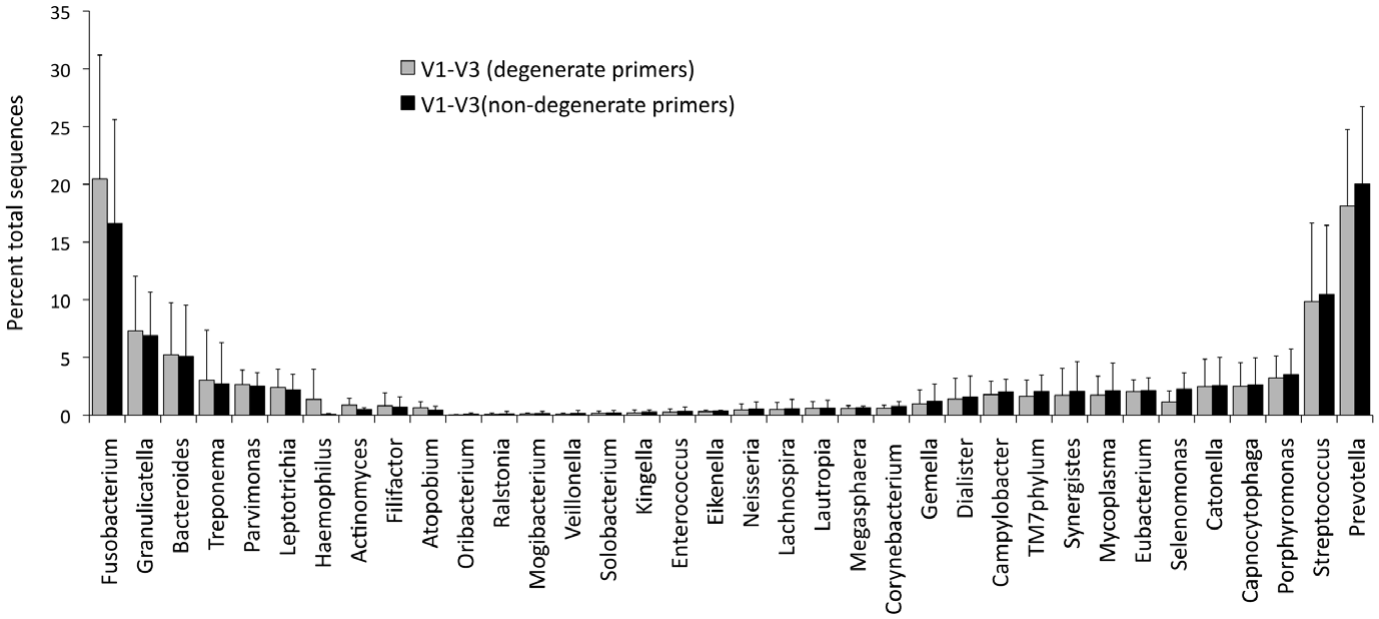
Distribution of sequences generated by degenerate and non-degenerate primers by genus. Percent mean abundances and standard deviations are shown. Genera are arranged in a gradient such that those predominant in the degenerate community are arranged on the left. There were no differences between the two communities in the relative abundance of any genus (p>0.05, 2-sample t-test on transformed variable).

Figure 3 shows the distribution by genus of sequences generated by degenerate and non-degenerate primer pairs targeted to the V1–V3 region. There were no differences between the two groups (p>0.05, 2-sample t-test on transformed variable).

Table 2 shows the relative abundance of genera in sequences obtained by pyrosequencing different target regions. Genera accounting for 0.1% of total pyrosequences are shown. Overall, greater numbers of differences were detected in the levels of genera between the V1–V3 and V7–V9 regions (p<0.05, 2-sample t-test on transformed variable). The regions targeted significantly influenced community profiles generated by pyrosequencing. The genera *Prevotella*, *Fusobacterium*, *Streptococcus*, *Granulicatella*, *Bacteroides*, *Porphyromonas* and *Treponema* formed 65% of the community when the V1–V3 region was targeted, while *Streptococcus*, *Treponema*, *Prevotella*, *Eubacterium*, *Porphyromonas*, *Campylobacter* and *Enterococcus* accounted for the same abundance in the community generated by V4–V6 primers, and 65% of the V7–V9 community was formed by *Veillonella*, *Streptococcus*, *Eubacterium*, *Enterococcus*, *Treponema*, *Catonella* and *Selenomonas*. Among the predominant genera, *Fusobacteria* were not detected in any of the samples by the V7–V9 primers, while the V4–V6 primers did not detect the *Selenomonads*, *Mycoplasma*, or *TM7 phylum* in any sample (p<0.05, Fisher's exact test).

**Table 2:** Relative abundances of genera in pyrotag sequences.

Genus	Percent total pyrotags	Percent abundance (mean ± standard deviation)		
		V1–V3	V4–V6	V7–V9
Streptococcus (A,B)	15.0	8.3±3.1	25.2±4.3	11.5±8.0
Prevotella ( A,B,C)	11.5	23.1±5.9	8.2±5.4	3.3±1.9
Fusobacterium (A,C)	7.3	18.3±8.3	3.6±2.0	0.0±0.0
Treponema(A)	7.3	1.8±4.2	12.2±3.3	7.8±10.2
Eubacterium ( C )	6.6	1.9±1.1	5.2±4.2	12.6±5.1
Enterococcus ( C )	5.3	0.3±0.4	5.3±2.4	10.3±5.0
Veillonella (B,C)	5.0	0.3±0.2	1.5±0.1	13.1±6.6
Selenomonas (B)	3.5	4.2±2.1	0.0±0.0	6.3±2.2
Granulicatella (A,C)	3.5	6.9±3.8	1.3±1.8	2.2±1.8
Dialister	3.4	1.6±1.8	3.1±0.4	5.4±7.1
Parvimonas (B)	3.4	2.6±1.2	1.2±0.6	6.3±2.6
Porphyromonas (B)	3.2	3.5±2.2	5.8±1.2	0.2±0.1
Campylobacter (B)	3.1	2.1±1.1	6.2±1.2	1.0±0.7
Catonella	3.0	1.9±2.4	1.2±2.2	5.9±6.4
Bacteroides ( C )	3.0	5.1±4.4	3.5±1.1	0.3±0.3
Synergistes (B)	2.2	2.1±2.6	4.3±0.9	0.3±0.3
Neisseria (A)	2.0	0.5±0.6	3.4±1.8	2.1±1.3
Capnocytophaga	1.7	2.6±2.3	1.3±1.8	1.1±1.1
Unclassified Bacteroidales (A, B)	1.6	1.1±0.3	3.4±1.4	0.4±0.7
Filifactor	1.5	0.7±0.9	1.9±0.3	1.8±2.3
Gemella	1.4	1.2±1.5	0.8±0.6	2.2±1.9
Unclassified Veillonellaceae ( C )	1.2	0.5±0.6	0.9±0.3	2.1±2.5
Megasphaera	1.0	0.7±0.1	0.5±0.1	1.8±0.6
Leptotrichia ( C )	1.0	2.2±1.3	0.5±0.1	0.3±0.4
TM7 phylum (A,C)	0.8	2.4±1.4	0.0±0.0	0.0±0.0
Mycoplasma (A,C)	0.7	1.9±2.4	0.0±0.0	0.1±0.1
Hemophilus	0.5	0.1±0.1	0.02±0.02	1.4±2.6
Lautropia	0.5	0.6±0.7	0.5±0.2	0.4±0.4
Corynebacterium	0.5	0.8±0.4	0.01±0.2	0.6±0.2
Arthrobacter	0.4	0.1±0	0±0	1.1±0.3
Actinomyces	0.3	0.5±0.2	0.02±0.2	0.5±0.2
Oribacterium	0.3	0.1±0.1	0.2±1.1	0.7±1.4
Kingella	0.3	0.3±0.2	0.5±0.1	0.0±0.0
Unclassified Clostridiales	0.2	0.3±0.4	0.1±0.01	0.3±0.2
Atopobium	0.2	0.4±0.3	0.1±0.2	0.2±0.2
Eikenella	0.2	0.4±0.1	0.2±0.2	0.0±0.0
Unclassified Lachnospiraceae	0.2	0.5±0.3	0.01±0.02	0.04±0.01
Lactococcus	0.2	0.1±0.1	0.0±0.0	0.4±0.3
Desulfobulbus	0.1	0.1±0.1	0.0±0.0	0.3±0.3
Ralstonia	0.1	0.1±0.2	0.0±0.0	0.2±0.2
Solobacterium	0.1	0.2±0.2	0.0±0.0	0.0±0.0

Percent mean abundances (and standard deviations) of genera in the 3 pyrotag and Sanger sequence communities are shown, arranged in order of decreasing overall prevalence. Alphabets in parentheses indicate statistically significant differences between groups (p<0.05, 2-sample t-test on transformed variable). A- significant difference between V1–V3 & V4–V6, B- significant difference between V1–V3 & V7–V9, C- significant difference between V4–V6 & V7–V9 (2-sample t-test on transformed variable).

Table 3 shows the relative abundance of genera obtained by concatenating data from pairs of target regions or by combining all three regions to provide near-full-length coverage of the 16S gene. Relative abundances of the same genera in near-full-length Sanger sequences are also shown for comparison. To arrive at these results, the subset pyrotag dataset was compared to an equivalent number of Sanger sequences from each sample. Concatenating data from V1–V3 and V7–V9 regions demonstrated the greatest similarity to Sanger data as well as to the averages of all 3 regions.

**Table 3:** Relative abundances of genera in Sanger and concatenated pyrotag datasets.

Genus	Average abundance (percentage)				
	V1–V3 & V4–V6	V4–V6 & V7–V9	V1–V3 & V7–V9	Sanger	V1–V3, V4–V6 & V7–V9
Streptococcus	11.7	14.3	16.2	17.8	14.1
Eubacterium	2.5	8.4	7.3	6.2	6.1
Veillonella	1.9	3.8	11.9	10.9	5.9
Treponema	6.1	4.6	4.8	4.6	5.2
Selenomonas	4.2	3.7	7.2	8.6	5
Catonella	1.6	7.6	4.3	5.9	4.5
Bacteroides	7.8	1.9	2.7	1.3	4.1
Fusobacterium	5.4	4.1	1.3	0.8	3.6
Granulicatella	4.1	1.8	4.6	2.2	3.5
Parvimonas	1.9	3.4	4.4	7.1	3.4
Dialister	2.4	4.3	3.5	3.2	3.4
Prevotella	3.6	4.2	2.1	1.3	3.3
Porphyromonas	4.7	3	1.9	2.1	3.2
Campylobacter	4.2	3.6	1.6	11.8	3.1
Gemella	4.7	1.5	2.2	3.6	2.8
Unclassified Bacteroidales	4.3	1.9	0.8	1.9	2.3
Synergistes	3.2	2.3	1.2	1.0	2.2
Enterococcus	0.6	3.2	2.7	0.0	2.2
Neisseria	2	2.8	1.3	0.5	2
Megasphaera	3.2	1.2	1.3	2.3	1.9
Capnocytophaga	2	1.2	1.9	1.9	1.7
Filifactor	1.3	1.9	1.3	2.1	1.5
Unclassified Veillonellaceae	0.3	2.3	0.6	0.2	1.1
Leptotrichia	1.4	0.4	1.3	0.0	1
Desulfobulbus	2.3	0.2	0.1	0.0	0.8
Lautropia	0.6	0.5	0.2	0.0	0.4
Corynebacterium	0.4	0.3	0.2	0.0	0.3
Actinomyces	0.3	0.3	0.3	0.2	0.3
Atopobium	0.3	0.2	0.3	0.5	0.2
Unclassified Clostridiales	0.2	0.2	0.3	0.0	0.2
TM7 phylum	0.2	0.0	0.4	0.0	0.2
Eikenella	0.3	0.1	0.2	0.6	0.2
Oribacterium	0.0	0.5	0	0.0	0.2
Arthrobacter	0.4	0.0	0.0	0.0	0.1
Kingella	0.0	0.3	0.0	0.0	0.1
Mycoplasma	0.0	0.0	0.0	0.0	0.0
Lactococcus	0.0	0.0	0.0	0.0	0.0
Ralstonia	0.0	0.0	0.0	0.0	0.0
Solobacterium	0.0	0.0	0.0	0.0	0.0
Hemophilus	0.0	0.0	0.0	0.0	0.0
Unclassified Lachnospiraceae	0.0	0.0	0.0	0.0	0.0

## Discussion

It has been shown that sequences of 500–700 bp are required for phylogenetic discrimination at the species levels [9], [29]. However, previous reports have been equivocal on the level of community coverage achieved using the different hypervariable regions. While several investigations support using the V1, V2 and V3 regions for deep sequencing [17], others suggest that these regions overestimate species richness and promote the V4–V6 region as the most appropriate [19]. Yet others have demonstrated that V7–V8 fragments achieve representational characterization of a community [30]. Our previous investigations with Sanger sequencing have revealed that the subgingival microflora associated with periodontitis in smokers is extremely diverse, with several rare species/phylotypes [*27*]. Hence, plaque samples were collected and pooled from deep sites of current smokers with moderate to severe periodontitis to examine the extent to which primer design affects the community fingerprint of a highly complex and taxonomically heterogeneous microbial population. Using an adequately powered clinical study design to enable statistical analyses allowed an in-depth comparison of the community profiles generated by the different primer sets.

The Shannon Diversity index incorporates both the number of species (species richness) as well as the proportion of each species (species evenness) into a single value [31]. Thus, while a value of zero necessarily represents a mono-species community, a higher value may result either from the presence of several species at varying levels or from equitable distribution of a few species. Hence, the Equitability index is used to elucidate the relative contributions of species richness and evenness to the Diversity index. Pyrotag communities demonstrated similar diversity to the Sanger community, however, were significantly less equitable (Figure 1), suggesting that greater species richness contributed to the diversity. The increased species richness was apparent in both rare and abundant taxa (Figure 2). This is in contrast to previous investigations; which have suggested that pyrosequencing overestimates community diversity by overestimating the number of rare taxa [10], [32]. A single-step PCR with low cycle numbers and a high fidelity, proofreading polymerase were utilized in this study; and it is possible that this minimized over-representation of rare taxa in the present investigation. No differences were apparent in the number of rare and abundant taxa between the different hypervariable regions; suggesting that targeting a specific region for pyrosequencing does not affect species richness. Taken together, it appears that selecting a specific region for pyrosequencing is not a source of bias in the diversity of the resulting community or in the number of taxa detected.

Out of the four primer pairs selected, two pairs targeted the same region (V1–V3), one pair containing degenerate sequences and the other non-degenerate. Fragments encompassing the V1–V3 region have been the most common targets for both Sanger sequencing and pyrosequencing; and both non-degenerate and degenerate primers have been used to amplify this region [2], [6], [7], [8], [9], [33]. It has previously been suggested that inclusion of degenerate sequences improves the “universality” of primers (reviewed by Baker et al [15]), however, our data does not support a role for primer degeneracy in improving community coverage. This is in concordance with previous investigations that have reported no effect of primer degeneracy on profiles of naturally occurring microbial communities [34]. Although degenerate primers, by virtue of their lowered specificity, may amplify larger number of taxa within a community, it has been shown that this effect is magnified when large PCR cycle numbers are used [35]. The present investigation used 22 cycles to amplification to ensure representational amplification of the community template, and it is possible that the low cycle numbers precluded a possible influence by degenerate primers.

Our data suggest that the hypervariable region targeted for sequencing plays a critical role in influencing the composition of pyrotag communities. Previous investigations have reported that amplicon size and PCR kinetics may be a source of sequencing bias [36], [37]. To overcome this in the present study, sequencing primers were carefully selected to generate similar amplicon sizes (∼500 bp for V1–V3 amplicons, ∼550 bp for V4–V6 amplicons and ∼470 bp for V7–V9 amplicons). Identical PCR cycling conditions were also utilized for all primer sets, thereby reducing the possibility of bias from this source. Using a single pyrosequencing run to generate all sequences further reduced bias due to PCR and sequencing kinetics. Thus, the observed differences could not be attributed to these variables. It is especially striking that even though these samples were derived from sites with severe disease, the V7–V9 communities were dominated by *Veillonella* and the V4–V6 communities by *Streptococci* (Table 2), genera that have been previously associated with periodontal health [33]. Similarly, *Treponema*, a disease-associated genus; was found in high numbers in the V4–V6 and V7–V9 communities; while other disease-associated genera, for example, *Prevotella*, *Porphyromonas*, and *Bacteroides* were predominant in V1–V3 communities derived from the same run. *Fusobacteria* were undetected by the V7–V9 primers while forming nearly 19% of the V1–V3 community. Similarly, the *Selenomonads* were not detectable by the V4–V6 primers, while forming 6% of the V7–V9 community. Concatenated data from V1–V3 and V7–V9 regions resulted in community profiles that did not significantly differ from Sanger sequences or full-length pyrosequences for the predominant genera, while averages of the other two regions did not yield similar results (Table 3). It is also noteworthy that the greatest differences were observed in the community fingerprints generated by these two primer sets. The mechanism causing this difference is not clear and warrants further investigation. It could be hypothesized that presence and nature of secondary structures within the target regions as well as the GC ratios of the resultant fragments may have contributed to the differences. It is known that the V1, V4 and V7 regions exhibit differences in the number of stems as well as in nucleotide variations within these stems [38], [39], and while is possible that differential amplification efficiencies contributed to the compositional differences, it is not within the scope of this study to test this hypothesis. It has been shown that higher GC ratios result in higher amplification efficiencies [35], thereby altering PCR kinetics, with over-amplification of rare members and under-representation of dominant species [40]. In the present investigation, however, the GC ratios of the different amplicons were very similar; therefore, the observed discrepancies could not be attributable to this variable.

In summary, the hypervariable region targeted by the primer plays a critical role in determining the profile of a largely uncultivated, complex microbial community generated by pyrosequencing. This effect is significant, with the presence of certain dominant community members being masked and others being under-represented with different primer sets; thereby providing a critical source of error in microbial ecological studies. However, averaging the community fingerprints generated by V1–V3 and V7–V9 primers provides results similar to Sanger sequencing, while allowing a significantly greater depth of coverage than is possible with Sanger sequencing. It is therefore important to use primers targeted to these two regions of the 16S rRNA gene in all deep-sequencing efforts to characterize heterogeneous microbial communities.
